# Molecular Characterization, Antibiotic Resistance, and Biofilm Formation of *Escherichia coli* Isolated from Commercial Broilers from Four Chinese Provinces

**DOI:** 10.3390/microorganisms13051017

**Published:** 2025-04-28

**Authors:** Saqib Nawaz, Muhammad Shoaib, Cuiqin Huang, Wei Jiang, Yinli Bao, Xiuyi Wu, Lianhua Nie, Wenyan Fan, Zhihao Wang, Zhaoguo Chen, Huifang Yin, Xiangan Han

**Affiliations:** 1 Shanghai Veterinary Research Institute, Chinese Academy of Agricultural Sciences, Shanghai 200241, China; nawazsaqib143@gmail.com (S.N.); jiangweijw99@163.com (W.J.); w1910160233@163.com (X.W.); 15110991273@163.com (L.N.); 15938272357@163.com (W.F.); wangzhihao2048@163.com (Z.W.); zhaoguochen@shvri.ac.cn (Z.C.); 2 Jiangsu Co-Innovation Center for Prevention and Control of Important Animal Infectious Diseases and Zoonoses, College of Veterinary Medicine, Yangzhou University, Yangzhou 225009, China; shoaibsinko8@gmail.com; 3 Engineering Research Center for the Prevention and Control of Animal Original Zoonosis, Fujian Province, College of Life Science, Longyan University, Longyan 364012, China; cuiqinh@126.com (C.H.); 82019007@lyun.edu.cn (Y.B.)

**Keywords:** *Escherichia coli*, APEC, virulence genes, serotypes, biofilm, antibiotic resistance

## Abstract

*Escherichia coli* (*E. coli*) represents a significant etiological agent of colibacillosis in poultry, resulting in considerable economic losses for the global poultry sector. The present study aimed to determine molecular characterization, antibiotic resistance, and biofilm formation of *E. coli* strains isolated from diseased broilers from four provinces of China. A total of 200 tissue samples were collected from the intestine, liver, crop, heart, and spleen and processed for microbiological examination. Molecular detection of *E. coli* strains, virulence genes, and serotypes was performed using polymerase chain reaction (PCR). Antibiotic susceptibility testing and biofilm formation were assessed using disk diffusion and 96-well microtiter plate assays. The study retrieved 68% (136/200) of *E. coli* strains from collected samples. Most of the *E. coli* strains were resistant to enrofloxacin (56%), followed by cefepime (54%), amoxicillin/clavulanate (52%), streptomycin (50%), ampicillin (48%), clindamycin (47%), kanamycin (41%), polymyxin B (37%), tetracycline (35%), sulfamethoxazole/trimethoprim (33%), ceftazidime (31%), meropenem (4.7%), and florfenicol (2.9%). Similarly, the *E. coli* strains tested positive for at least one virulence gene and specific serotypes. Among these, O145 was the most prevalent serotype, identified in 22 isolates (16.2%), followed by O8 (12.5%), O102 (11.8%), and O9 (11.0%). The *tsh* gene (10.2%) was the most prevalent virulence gene. This study found that 47.1% of *E. coli* strains were biofilm-producing, with 62.5% exhibiting weak biofilm production, 29.7% mild biofilm production, and 7.8% strong biofilm production. Similarly, 24.2% of the *E. coli* strains were avian pathogenic *E. coli* strains due to the presence of five or more virulence genes, specifically *tsh*, *ompA*, *fimC*, *iss*, *fyuA*, and *astA*, in a single strain by multiplex PCR. The present study recommends continuous surveillance and effective control measures to reduce the burden of avian pathogenic *E. coli*-related infections in poultry.

## 1. Introduction

*Escherichia coli* (*E. coli*) is a ubiquitous bacterium that typically predominates in the gut microflora of humans, animals, and birds [1]. Apart from commensal *E. coli*, various pathogenic strains cause intestinal infections [2]. *E. coli* in poultry leads to colibacillosis, which is manifested as perihepatitis, pericarditis, air-sacculitis, salpingitis, and peritonitis, potentially progressing to septicemia and death [3]. This is one of the leading causes of mortality and morbidity in poultry, affecting all stages of production and being economically devastating to the industry [4]. It is estimated that at least 30% of the commercial flocks in the United States are affected by colibacillosis at any point, resulting in multi-billion-dollar losses to the poultry industry annually [5,6]. *E. coli* can infect poultry through various routes. Oral and respiratory pathways are common routes that facilitate bacterial colonization of the gastrointestinal and respiratory tracts, allowing migration to internal organs and causing infection. Infected birds can transmit the bacteria to other birds by contaminating feed and water [3,4,7]. Previously, *E. coli* was regarded as a secondary pathogen that caused disease outbreaks with concurrent viral infections, improper management practices, or inadequate egg hygiene. However, recent research has identified its potential role as a primary pathogen that causes severe disease and high mortality in the absence of stressors [8,9,10].

The pathogenic attributes of *E. coli* are facilitated by multiple virulence factors, such as adhesins, invasins, protectins, iron acquisition mechanisms, toxins, and plasmids [11]. These factors enable attachment, invasion, colonization, replication, damage to the host cells, and evasion of the host immune response [12,13]. An array of virulence-associated genes encodes these virulence factors, including *papC* (pilus associated with pyelonephritis) and *tsh* (temperature-sensitive hemagglutinin), which are responsible for adhesion, and *ibeA* (invasion of the brain endothelium protein A), which governs the invasion of the host system. Other genes, such as *iutA* (aerobactin siderophore receptor) and *iroN* (salmochelin and catecholate siderophore receptor), facilitate iron acquisition from body fluids. The *iss* (increased serum survival), *ompT*, and *ompA* (outer membrane protease) protect the host immune response. Furthermore, *astA* (heat-stable enterotoxin), *hlyE* (putative avian hemolysin), *iucD* (aerobactin synthesis), *vat* (vacuolating autotransporter toxin), *cvi*/*cva* (structural genes of colicin V operon), *fimC* (type 1 fimbriae (D-mannose-specific adhesin), and *fyuA* (ferric yersinia uptake) enable *E. coli* to produce toxins that damage the host’s tissues [14,15]. Several studies have identified combinations of different virulence genes to predict the disease-causing potential of *E. coli* strains [16,17]. Virulence genes also significantly contribute in facilitating the biofilm formation by these bacterial species [18].

Biofilm formation can serve as a key survival strategy for bacteria, enabling them to survive within host cells while enhancing their antibiotic resistance. The biofilm is characterized by a polymeric matrix that adheres to the surfaces of bacterial cells [19] and provides numerous benefits to bacteria, such as structural integrity, enhanced adhesion through bacterial adhesins, and protection against immune responses [20]. Moreover, the high diversity of *E. coli* strains makes it challenging to ensure the accuracy of these predictors, and thus hinders the effective diagnosis, treatment, and prevention of *E. coli* infections in poultry [21]. The high diversity of *E. coli* strains is further evident in the number of serotypes established. Serotyping is vital for unraveling *E. coli* virulence mechanisms [22]. O (lipopolysaccharide) and H types (flagellar antigen) have been widely used to classify *E. coli* strains. Some of the O serogroups associated with *E. coli* strains in poultry are O1, O2, O8, O9, O18, O21 [23,24,25,26,27], O78, O102, O128, and O145 [28,29,30]. Serotyping, along with biofilm and virulence detection, is a better predictor of the virulence potential of *E. coli* [31].

*E. coli* has been reported to resist various antibiotics, such as tetracyclines, sulphonamides, and aminoglycosides, commonly used in the poultry industry to treat *E. coli* infections [3]. Antimicrobial resistance and virulence genes are often associated with plasmids that facilitate their transmission between bacteria, and thus need to be continuously monitored [32]. The present study investigated the molecular characterization, antibiotic resistance, and biofilm formation of *E. coli* strains isolated from tissue samples of diseased broiler birds from four provinces of China.

## 2. Materials and Methods

### 2.1. Sample Collection

Samples from four provinces recognized for broiler production were obtained to ensure comprehensive geographical representation and account for potential variations in *E. coli* strains. These samples may not accurately represent the entire population of affected broilers in these areas. They were chosen from farms with documented histories of colibacillosis outbreaks to enhance the chances of isolating pertinent strains. The samples were taken from broilers exhibiting specific clinical signs of colibacillosis, such as coughing, sneezing, nasal discharge, labored breathing, gasping, yellowish diarrhea, uncoordinated movements, and joint swelling. Characteristic post-mortem lesions, including fibrinous pericarditis, peritonitis, air sacculitis, and hepatomegaly, supported the suspected diagnosis. A licensed veterinarian performed a clinical evaluation to validate the suspicion of colibacillosis before sampling. Only cases detected with distinct clinical symptoms and post-mortem findings of colibacillosis were included in this study, and systemic infections were ruled out through clinical and post-mortem findings. A total of 200 tissue samples of liver (*n* = 20), spleen (*n* = 20), heart (*n* = 20), intestine (*n* = 120), and crop (n = 20) of colibacillosis-suspected broilers were received from October 2023 to October 2024 at Shanghai Veterinary Research Institute from commercial broiler farms in Liaoning (*n* = 50), Shandong (*n* = 50), Fujian (*n* = 50), and Xinjiang (*n* = 50) provinces of China. Each province received 5, 5, 5, 30, and 5 samples of liver, spleen, heart, intestine, and crop, respectively. Organ samples were collected from only those birds that had shown symptoms on post-mortem examination. All of the samples were labeled and processed immediately for bacteriological examination.

### 2.2. Bacterial Isolation and Primary Identification

A tissue sample of approximately 1 g was collected and homogenized in 1 mL of 0.9% sterile normal saline. A small volume of 100 μL was cultured in 3 mL of Luria-Bertani (LB) broth and incubated at 37 °C for 4 to 6 h to enrich the culture. After enrichment, a small inoculum of 100 μL was spread on MacConkey agar plates and incubated at 37 °C for 24 h in an aerobic incubator (Galaxy 48S, New Brunswick, and Eppendorf Company, Enfield, CT, USA). From each organ, 1 to 2 distinct pink colonies exhibiting morphological characteristics typical of *E. coli* were selected and streaked again on MacConkey agar plates until pure colonies were obtained. Following purification, a single purified colony was picked and streaked on Eosin Methylene Blue (EMB) agar for further phenotypic confirmation of *E. coli*. The metallic green sheen colonies on EMB agar were picked and cultured again in LB broth under the same incubation conditions to prepare a 20% glycerol stock and for DNA extraction.

### 2.3. Molecular Identification of E. coli

Bacterial DNA extraction was performed using the Universal DNA Extraction and Purification Kit (Tiangen, Beijing, China) following the manufacturer’s guidelines [33]. Polymerase chain reaction (PCR) was performed to detect *E. coli* using specific primers targeting the *phoA* gene (*phoA*-F 5′-GAAACAAAGCACTATTGCAC-3′, *phoA*-R 5′-GGCTTTTGTCACAGGGGTAA-3′) of *E. coli* from an earlier study [27]. Briefly, a 20 µL reaction mixture was prepared, which included primeMix 10 μL, phoA-F 1 μL, phoA-R 1 μL, DNA-free water 7 μL, and DNA template 1 μL. The reaction was carried out with an initial denaturation step at 94 °C for 5 min, followed by 30 cycles of denaturation at 94 °C for 30 s, annealing at 58 °C for 30 s, and extension at 72 °C for 30 s. The final amplification step lasted for 10 min at 72 °C. After amplification, the PCR products were run on a 1% agarose gel at 180 V and a 400-mA current for 25 min. The PCR-positive samples were sent for Sanger sequencing for final confirmation.

### 2.4. Molecular Identification of E. coli Serotypes and Virulence Genes

All PCR and sequencing confirmed that *E. coli* was examined for the presence of fifteen virulence genes, including *iroN*, *iutA*, *iss*, *ompT*, *ompA*, *hlyE*, *astA*, *papC*, *tsh*, *ibeA*, *iucD*, *vat*, *cvi/cva*, *fimC*, and *fyuA* (Eurofins Genomics LLC, Louisville, KY, USA). Conventional PCR was employed to detect virulence genes using specific gene primers at varying annealing temperatures (listed in Table 1). Briefly, a 20 µL reaction mixture was prepared, consisting of primeMix 10 μL, 1 μL of forward and reverse primers of each gene, 7 μL of DNA-free water, and 1 μL of DNA template. The reaction commenced with initial denaturation at 94 °C for 5 min, followed by 30 cycles of denaturation at 94 °C for 30 s, at the different annealing temperatures specified in Table 1 for 30 s, and amplification at 72 °C for 30 s. The final amplification step lasted for 10 min at 72 °C. All PCR products were analyzed through agarose gel electrophoresis using a 1% agarose gel (Bio-Rad Laboratories, Inc., Madrid, Spain) and visualized under UV light [34]. Additionally, these *E. coli* isolates were screened for the presence of O1, O2, O8, O9, O18, O21, O78, O102, O128, and O145 serotypes by PCR [25] that encode for different virulence genes such as *iroN*, *iutA*, *iss*, *ompT*, *ompA, hlyE*, *astA*, *papC*, *tsh*, *ibeA*, *iucD*, *vat*, *cvi/cva*, *fimC*, and *fyuA*. The primers for the *E. coli* serotypes and virulence genes were designed for serotyping and are shown in Table 1.

### 2.5. Antibiotic Susceptibility of E. coli Isolates

All *E. coli*-positive isolates were analyzed for antibiotic susceptibility testing using the agar disc diffusion assay, consistent with the modified Kirby–Bauer disc diffusion technique. The multidisc dispenser (Abtek Biologicals Ltd., Liverpool, UK), containing various antibiotic discs, was aseptically placed on bacteria-inoculated Mueller–Hinton (MH) agar plates and incubated at 37 °C for 18–20 h. After incubation, the plates were examined for the zone of inhibition. The diameter of the inhibition zones produced by each antibiotic disk was measured using a calibrated Vernier caliper and interpreted according to the Clinical and Laboratory Standards Institute Standards. Antibiotic susceptibility testing was conducted using the following antibiotics from different classes: Lincosamides (Clindamycin, CLI-2 μg), Carbapenems (Meropenem, MEM-10 μg), Cephalosporins (Ceftazidime, CAZ-30 μg; Cefepime, FEP-30 μg), Amphiphenicols (Florfenicol, FFC-30 μg), Penicillin (Ampicillin, AMP-10 μg; Amoxicillin/Clavulanate, AMC 20 μg/10 μg), Aminoglycosides (Kanamycin, KAN-15 μg; Streptomycin, STR-10 μg), Tetracycline (Tetracycline, TCY-30 μg), Polypeptide (Polymyxin B, PB-30 μg), Co-trimoxazole (Sulfamethoxazole/Trimethoprim, SXT-25 μg), and Fluoroquinolone (Enrofloxacin, ENR-5 μg). The data were also analyzed for multidrug-resistant (MDR) strains. The resistance of *E. coli* isolates to ≥3 different antibiotic classes was referred to as MDR [38].

### 2.6. Biofilm Formation Assay of E. coli Isolates

Bacterial biofilms were measured in 96-well polyvinyl chloride (PVC) microplates using a crystal violet assay, as described previously [39]. Each strain’s overnight culture was diluted 1:100 in Mueller–Hinton Broth (MH Broth) (Thermo Scientific, Oxoid, UK), supplemented with 0.5% glucose (*w*/*v*), and inoculated into 96-well PVC microplates. Biofilms were allowed to develop at 37 °C for 24 h. The cells that adhered to the microtiter wells were stained with crystal violet (0.1%, *w*/*v*), subsequently solubilized with 95% ethanol, and the optical density (OD) was measured at 620 nm using a spectrophotometer. The *E. coli* DH5α (C) was a negative control strain. Biofilm formation capacity was assessed by OD evaluation: OD of the strain the same as or lower than C (negative control), weakly positive (C < strain ≤ 2C), mild positive (2C < strain ≤ 4C), and strongly positive (4C < strain) [40].

### 2.7. Pathogenicity Assay of E. coli Isolates for Avian Pathogenic Escherichia coli (APEC) Strains

*E. coli*-positive isolates were screened for key virulence genes (*ompA*, *astA*, *iss*, *papC*, *tsh*, *ibeA*, *fimC*, and *fyuA*) using a well-established multiplex PCR assay, and those harboring ≥5 genes were classified as avian pathogenic *E. coli* (APEC) strains, following the criteria of Johnson et al. [35] and Ewers et al. [41]. Primer sequences and gene definitions are shown in Table 1. Reactions were performed in a 25 μL volume containing 2.5 μL of 10x PCR buffer, 0.4 μL of 50 mm MgCl_2_, 1.25 μL of dNTP (10 μM) Pool, 2 U Taq DNA polymerase, 0.075 μL (200 μM) of each primer, and 2 μL of DNA sample. The reaction conditions were as follows: the annealing temperature was adjusted to 58 °C, followed by 94 °C for 5 min. The reaction consisted of 30 cycles of 94 °C for 30 s, 63 °C for 30 s, and 68 °C for 10 min, with a final extension step at 72 °C for 10 min.

### 2.8. Statistical Analysis

The data were saved and processed in Microsoft Excel to analyze percentage prevalence using the formula:

$$Prevalence\left(\%\right)=\frac{No.\;of\;positive\;isolates}{Total\;isolates}\times 100$$

Descriptive statistics were used to analyze the antimicrobial susceptibility data. Moreover, a generalized linear model (GLM) with binomial distribution was used to assess differences in *E. coli* prevalence across different provinces and tissue types, accounting for unequal sample sizes. Additionally, post hoc pairwise comparisons with Bonferroni correction were applied to determine significant differences among groups. Analyses were performed using the stats model package in Python 3.12. A *p*-value < 0.05 was considered statistically significant and vice versa [17]. The graphs were made using GraphPad Prism version 8.2.1.

## 3. Results

### 3.1. Detection of E. coli isolates

**Figure 1:**
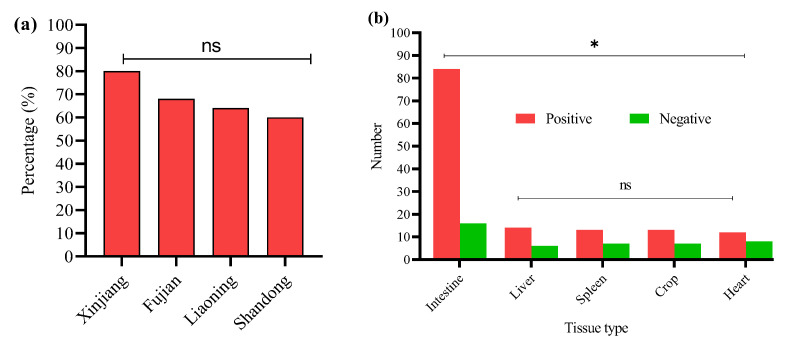
(**a**) Percentage of positive *E. coli* from four Chinese provinces. (**b**) Distribution of *E. coli* from different sample types. * Indicates a significant difference; ^ns^ indicates a non-significant difference.

In this study, 200 tissue samples were collected from broiler birds suspected of having colibacillosis across four provinces of China. The study found that 68% (136/200) of the total samples tested positive for *E. coli* via PCR (Supplementary Figure S1). The GLM analysis showed no statistically significant differences in *E. coli* prevalence among the provinces. The prevalence of *E. coli* was highest in Xinjiang (80%, 40/50), followed by Fujian (68%, 34/50), Liaoning (64%, 32/50), and Shandong (60%, 30/50) (Figure 1a); the logistic regression model did not reveal statistically significant differences between provinces (*p* > 0.05 for all comparisons). These results suggest that *E. coli* prevalence is relatively consistent across geographically diverse poultry farms in China when accounting for sample size variability (Table 2). The GLM model estimates the log-odds of *E. coli* positivity relative to Fujian.

*E. coli* was most frequently isolated from intestinal samples *n* = 84/120, with significantly lower detection in liver *n* = 14/20, spleen *n* = 13/20, crop *n* = 13/20, and heart *n* = 12/20 (Figure 1b). A GLM (binomial distribution) confirmed a significant effect of tissue type on *E. coli* prevalence (* *p* < 0.05), with intestinal isolates showing the highest likelihood of positivity. These findings indicate the intestine as the primary site of *E. coli* colonization in broilers, with potential systemic dissemination at later stages of infection. However, non-significant differences (^ns^ *p* > 0.05) were noted among the liver, spleen, crop, and heart.

### 3.2. Detection of O-Serotypes in E. coli-Positive Samples

**Figure 2:**
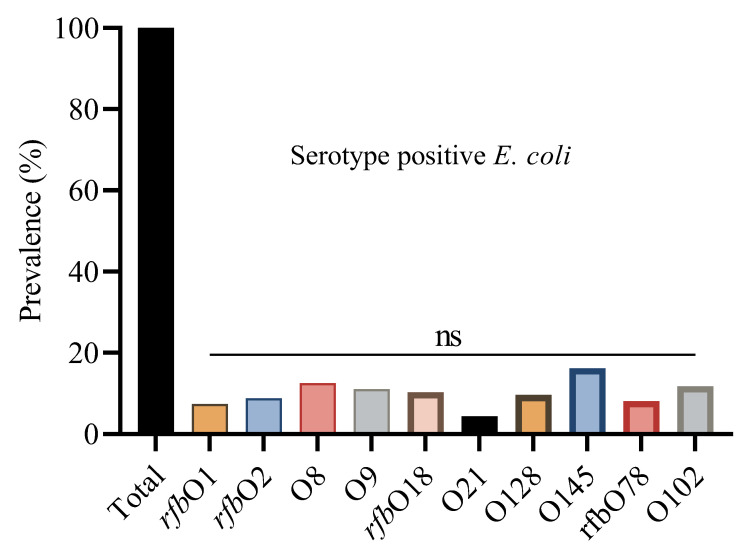
Overall percentage prevalence of *E. coli* serotypes. ^ns^ indicates a non-significant difference.

The prevalence of *E. coli* serotypes was detected by PCR (Supplementary Figure S2). Using a PCR-based method, we determined the O group of 136 *E. coli* isolates. The serotype prevalence showed that O145 was the most prevalent serotype, identified in 22 isolates (16.2%), followed by O8 (12.5%), O102 (11.8%), and O9 (11.0%). The O145 serotype was consistently found across all four regions, with the highest occurrence in Shandong (17.6%), and similar levels in Liaoning (15.6%), Fujian (16.7%), and Xinjiang (15%). Other frequently detected serotypes included *rfb*O18 (10.3%), *rfb*O2 (8.8%), *rfb*O78 (8.1%), and *rfb*O1 (7.4%). Some serotypes, like O128 and O21, were found in fewer isolates and were unevenly distributed across regions (Figure 2).

Statistical analysis showed non-significant differences (ns) in serotype distribution among the provinces. This suggests that these serotypes are geographically distributed and may reflect common circulating *E. coli* strains in Chinese broiler populations (Table 3).

### 3.3. Detection Rate of Virulence Genes in Positive O-Serotype Samples

This study analyzed fifteen virulence genes, i.e., *iroN*, *iutA*, *iss*, *ompT*, *ompA, hlyE*, *astA*, *papC*, *tsh*, *ibeA, iucD, vat, cvi/cva, fimC,* and *fyuA* in 136 positive serotype *E. coli* strains using PCR (Supplementary Figure S2). Overall, the positive serotype strains were found positive for at least one of the virulence genes, with *tsh* (10.2%) being the most prevalent, followed by *iss* (8.8%), *ompA, papC,* and *fyuA* (8% each), *astA, ibeA,* and *fimC* (7.3% each), *iutA, vat,* and *hlyE* (5.8% each), *iucD* (5.1%), *cvi/cva* and *iroN* (4.4%), and *ompT* (2.9%) (Table 4).

In Xinjiang, the prevalent virulence genes were *astA*, *iss*, and *ibeA*, each at 10%. Similarly, in Shandong, the prevalent genes were *tsh* and *fyuA*, each at 11.7%. In Liaoning, the prevalent gene was *tsh* (12.5%), while in Fujian, the prevalent genes identified were *papC*, *tsh*, *ibeA*, *iucD*, and *cvi/cva*, each at 10%.

### 3.4. Antibiotic Susceptibility Profile

**Figure 3:**
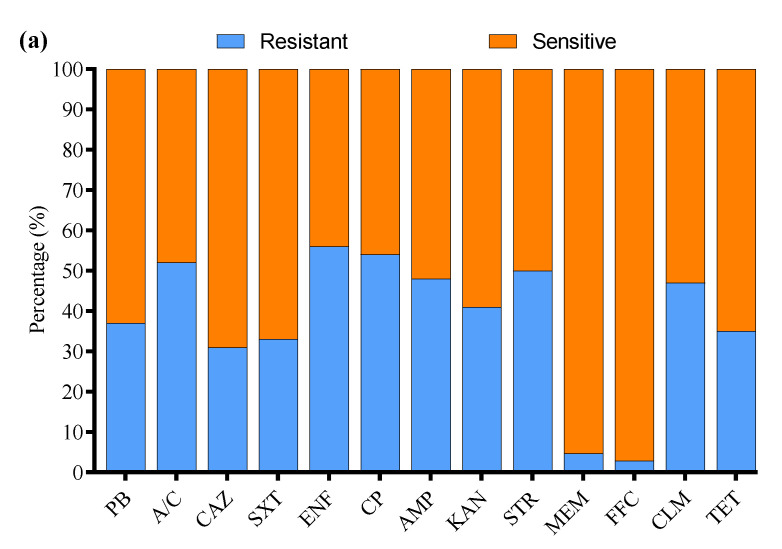

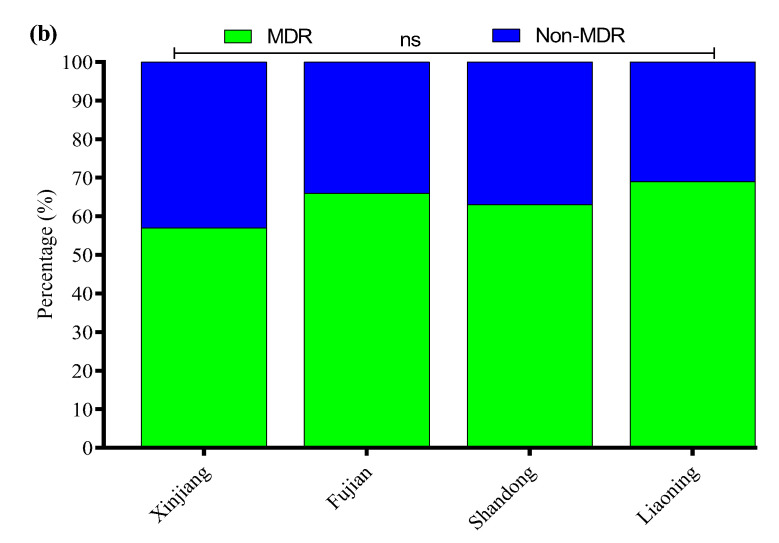
(**a**) Antibiotic susceptibility profile of *E. coli*-positive isolates. (**b**) Percentage prevalence of MDR *E. coli* isolates in different provinces of China. ^ns^ indicates a non-significant difference.

All 136 positive *E. coli* strains for O-serotypes and virulence genes were subjected to antibiotic susceptibility testing using the Kirby–Bauer disk diffusion assay (Supplementary Figure S3). Most of the *E. coli* strains were resistant to enrofloxacin (56%), followed by cefepime (54%), amoxicillin/clavulanate (52%), streptomycin (50%), ampicillin (48%), clindamycin (47%), kanamycin (41%), polymyxin B (37%), tetracycline (35%), sulfamethoxazole/trimethoprim (33%), ceftazidime (31%), meropenem (4.7%), and florfenicol (2.9%) (Figure 3a). Moreover, the MDR analysis showed a higher prevalence of MDR *E. coli* isolates in Liaoning (69%), compared to Fujian (66%), Shandong (63%), and Xinjiang (57%) (Figure 3b).

### 3.5. Biofilm Formation Ability

**Figure 4:**
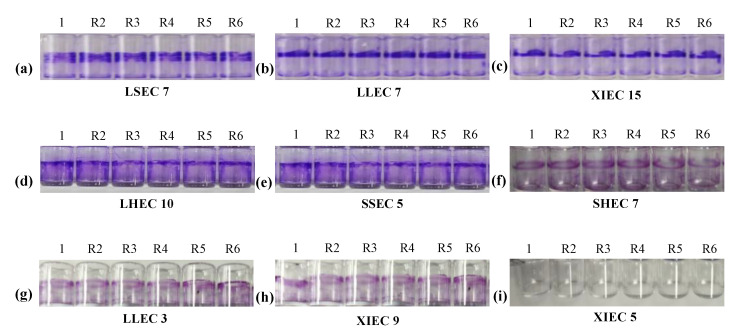
Biofilm formation of *E. coli* isolates: (**a**) Liaoning spleen *E. coli* (LSEC 7) strong biofilm, (**b**) Liaoning liver *E. coli* (LLEC 7) strong biofilm, (**c**) Xinjiang intestine *E. coli* (XIEC 15) strong biofilm, (**d**) Liaoning heart *E. coli* (LHEC 10) mild biofilm, (**e**) Shandong spleen *E. coli* (SSEC 5) mild biofilm, (**f**) Shandong heart *E. coli* (SHEC 7) weak biofilm, (**g**) Liaoning liver *E. coli* (LLEC 3) weak biofilm, (**h**) Xinjiang intestine *E. coli* (XIEC 9) weak biofilm, and (**i**) Xinjiang intestine *E. coli* (XIEC 5) Negative/No biofilm formation.

The biofilm-forming abilities of 136 positive O-serotypes and virulence genes of *E. coli* strains were evaluated using a crystal violet 96-well microtiter plate assay (Figure 4).

**Figure 5:**
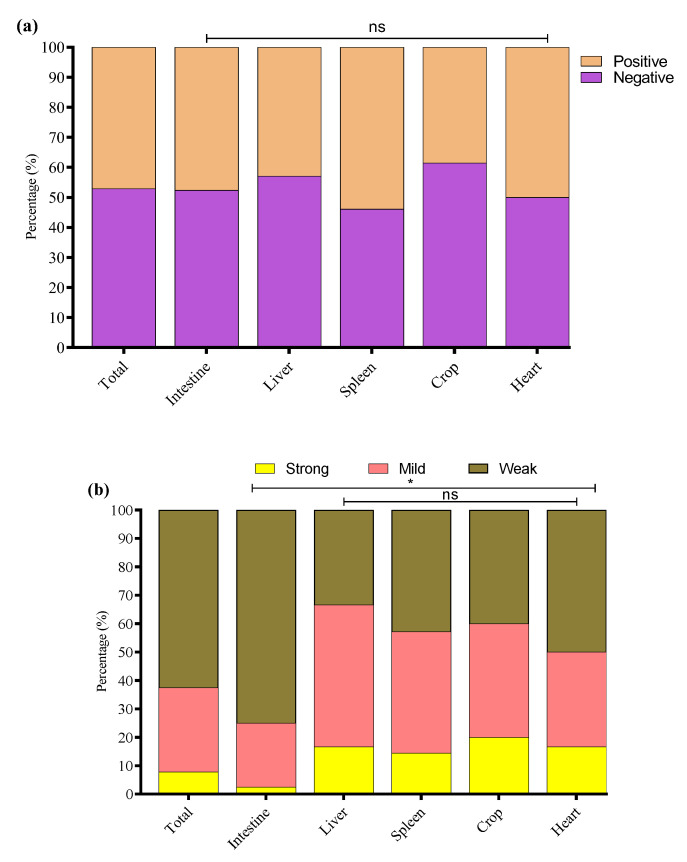
(**a**) Overall, biofilm-forming (positive) and non-biofilm-forming (negative) *E. coli* strains were isolated from different tissues. (**b**) Percentage of weak, mild, and strong biofilm-producing *E. coli* among biofilm-positive *E. coli* in various tissue samples. * Indicates a significant difference; ^ns^ indicates a non-significant difference.

The study noted that 64/136 (47.1%) *E. coli* strains were biofilm producers while 72/136 (52.9%) were non-biofilm producers. Among biofilm producers, 47.6% (40/84) were *E. coli* of intestinal origin, 42.9% (6/14) of liver origin, 53.9% (7/13) of spleen origin, 38.5% (5/13) of crop origin, and 50% (6/12) of heart origin (Figure 5a). Among the biofilm-producing *E. coli*, 62.5% (40/64) were weak, 29.7% (19/64) were mild, and 7.8% (5/64) were strong biofilm-producing *E. coli* (Figure 5b). To investigate the potential link between adhesion-related virulence genes and biofilm formation, we conducted a correlation analysis between the presence of *papC* and *tsh* genes and the intensity of biofilm formation, categorized as none, weak, mild, or strong. Our findings revealed a weak negative correlation between *papC* and biofilm formation score (r = −0.048), and a low positive correlation for *tsh* (r = 0.155). These results indicate that while *tsh* may have a minor association with increased biofilm production, neither gene alone strongly predicts biofilm formation in the tested strains. This suggests that biofilm formation in *E. coli* may be governed by multiple factors beyond *papC* and *tsh*, requiring further investigation into regulatory pathways and additional genetic contributors. The percentage distribution of weak, mild, and strong biofilm-producing *E. coli* from different tissues is presented in Figure 5b.

### 3.6. Detection of APEC Strains in E. coli Isolates

**Figure 6:**
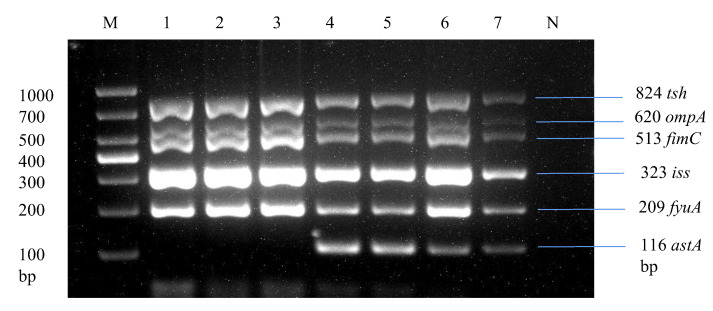
Development of a multiplex PCR for detecting APEC strains. Lane 1: Liaoning spleen APEC strain. Lane 2: Liaoning liver APEC strain. Lane 3: Xinjiang intestine APEC strain. Lane 1–3: Products of multiplex PCR containing *tsh*, *ompA*, *fimC*, *iss*, and *fyuA* virulence genes. Lane 4: Liaoning heart APEC strain. Lane 5: Shandong spleen APEC strain. Lane 6: Fujian intestine APEC strain. Lane 7: Fujian liver APEC strain. Lane 4–7: Products of multiplex PCR containing *tsh*, *ompA*, *fimC*, *iss*, *fyuA*, and *astA* virulence genes.

Through multiplex PCR, we analyzed strains by screening the most prevalent virulence genes across *E. coli* isolates, as shown in Table 3, including *ompA*, *astA*, *iss*, *papC*, *tsh*, *ibeA*, *fimC*, and *fyuA*. Overall, 24.2% (33/136) of the *E. coli* strains were found to be positive for 5 or 6 virulence genes, specifically *tsh*, *ompA*, *fimC*, *iss*, *fyuA*, and *astA*, in a single strain, as illustrated in Figure 6. These strains were classified as avian pathogenic *E. coli* strains due to the presence of five or more virulence genes of the total eight virulence genes in a single strain, which is the basic criterion for determining a pathogenic strain as per the standards of Johnson et al. [35] and Ewers et al. [41].

### 3.7. Antibiotic Susceptibility Profile of APEC Strains

**Figure 7:**
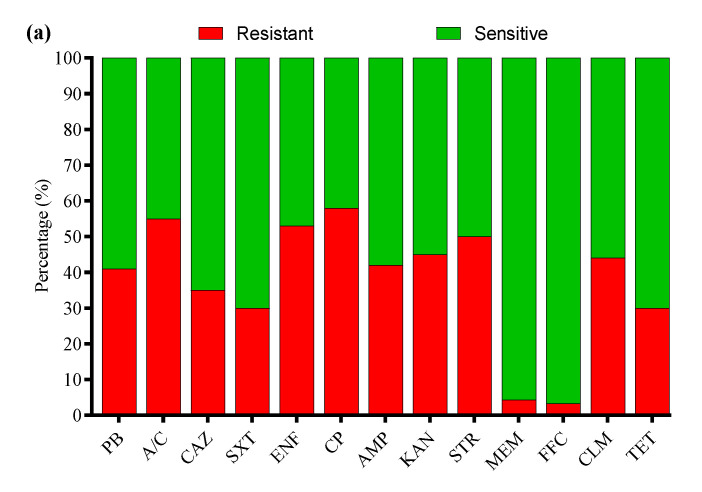

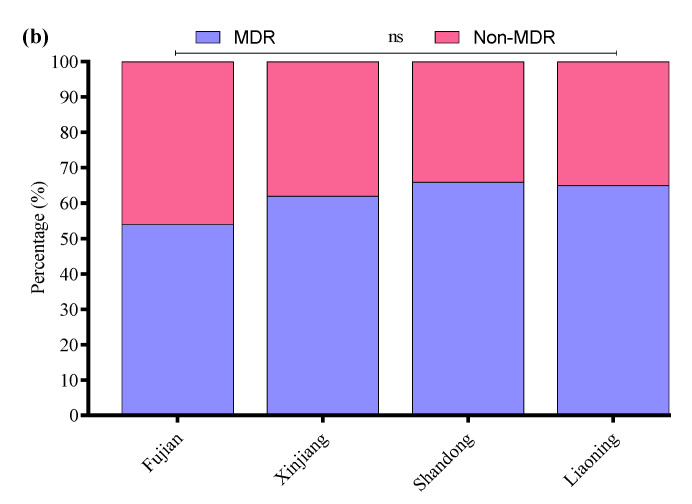
(**a**) Antibiotic susceptibility profile of APEC strains. (**b**) Percentage prevalence of MDR APEC strains in different provinces of China. ^ns^ indicates a non-significant difference.

All 33 APEC strains were subjected to AST using the Kirby–Bauer disk diffusion assay. Most of the APEC strains were resistant to cefepime (58%), followed by amoxicillin/clavulanate (55%), enrofloxacin (53%), streptomycin (50%), kanamycin (45%), clindamycin (44%), ampicillin (42%), polymyxin B (41%), ceftazidime (35%), tetracycline and sulfamethoxazole/trimethoprim (30% each), meropenem (4.3%), and florfenicol (3.3%) (Figure 7a). Moreover, the MDR analysis showed a higher prevalence of MDR APEC strains from Shandong (66%) compared to Liaoning (65%), Xinjiang (62%), and Fujian (54%) (Figure 7b).

### 3.8. Biofilm Formation of APEC Strains

**Figure 8:**
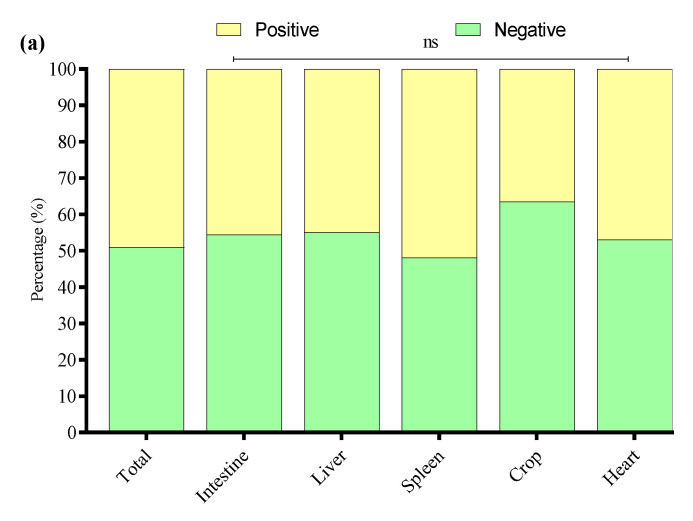

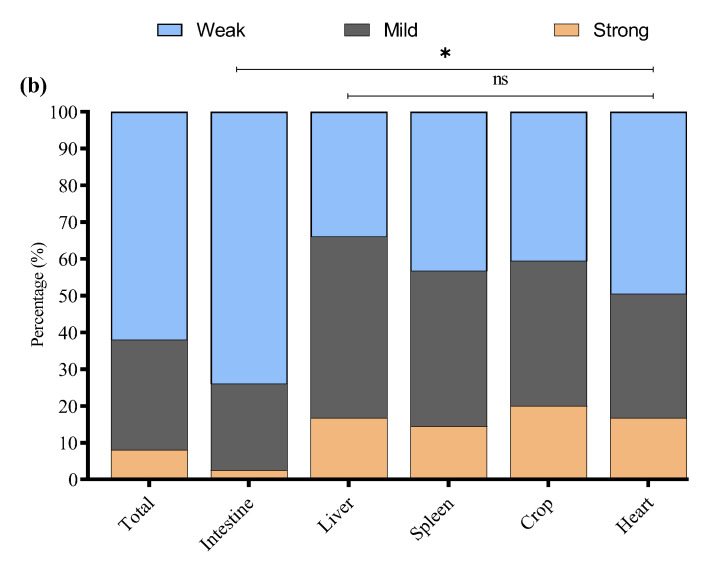
(**a**) Overall biofilm forming (positive) and non-biofilm forming (negative) by APEC strains isolated from different tissues. (**b**) Percentage of weak, mild, and strong biofilm-producing among biofilm-positive APEC strains in various tissue samples. * Indicates a significant difference; ^ns^ indicates a non-significant difference.

The biofilm-forming abilities of 33 APEC strains were evaluated through a crystal violet 96-well microtiter plate assay. The study noted that 49.1% of APEC strains were associated with biofilm production, while 50.9% were not. Among biofilm producers, 45.6% were of intestinal origin, 44.9% of liver origin, 51.9% of spleen origin, 36.5% of crop origin, and 47% of heart origin (Figure 8a). Among the biofilm-producing APEC strains, 62% were weak, 30% were mild, and 8% were strong biofilm producers. The percentage distribution of weak, mild, and strong biofilm-producing APEC strains across different tissues is presented in Figure 8b.

## 4. Discussion

*E. coli* is recognized as a prevalent etiological agent of Gram-negative infections [42]. Extraintestinal pathogenic *E. coli* (ExPEC) is classified as a facultative pathogen and encompasses several subtypes, including uropathogenic *E. coli* (UPEC), neonatal meningitis *E. coli* (NMEC), sepsis-associated *E. coli* (SEPEC), and avian pathogenic *E. coli* (APEC). Many studies reported the characteristics of *E. coli* isolates from layers worldwide [43,44]. However, there is limited information about the *E. coli* isolates from broiler birds. Recent studies have highlighted the importance of broilers as reservoirs for *E. coli* infection through vertical transmission to chicks and subsequent horizontal transmission between chicks [45,46]. Moreover, the evolving genetic diversity of *E. coli* strains requires continuous monitoring among all poultry species [47]. Strains of ExPEC are characterized by the presence of distinct virulence factors (VFs), which include adhesins, toxins, and iron acquisition mechanisms [11,48]. Numerous studies have explored the correlation between antimicrobial resistance and the presence of specific VFs with the capacity of ExPEC strains to form biofilms, which are implicated in urinary tract infections, bloodstream infections, and other extraintestinal conditions.

In this study, we characterize *E. coli* isolates from broilers with colibacillosis in Fujian, Liaoning, Shandong, and Xinjiang provinces, and also provide information on their genotypic-virulence properties. The prevalence of virulence genes tested in the present study is 68%. The genes encoding for temperature-sensitive hemagglutinin (*tsh*), outer membrane protease (*ompA*), type 1 fimbriae (D-mannose-specific adhesin) (*fimC*), increased serum survival (*iss*), ferric yersinia uptake (*fyuA*), and heat-stable enterotoxin (*astA*) were found to exhibit the highest prevalence among the isolates. The *tsh* and *iss* genes are associated with the ColV plasmid [49] and have been identified as genes more predominantly associated with highly avian pathogenic *E. coli* predictors. A similarly high prevalence of these genes was observed in *E. coli* isolated from broilers and broiler breeders with colibacillosis from different geographical locations, such as Canada [50], Brazil [51], Egypt [52], Korea [32], and the United States [10].

Similarly, the occurrence of virulence genes analyzed, *papC*, *hlyE*, *ibeA*, and *tsh*, was also less than the minimal predictors and was similar to that reported in *E. coli* from broilers in Nepal [36]. The gene encoding the mechanism for adhesion, *tsh,* was the lowest among the isolates and was comparable to that observed in *E. coli* isolated from cellulitis lesions in turkeys from Iowa, USA [33]. The current results indicate the virulence-defining nature of the minimal-predictor genes in *E. coli* isolates from broilers with colibacillosis. *E. coli* may be classified by somatic (O), capsular (K), and flagellar (H) antigens [53]. Pathogenicity is linked to distinct O-antigen serotypes, i.e., O1, O2, O8, O9, O18, O21, O78, O102, O128, and O145, which have been linked to avian pathogenic *E. coli* outbreaks, accounting for over 50% of reported cases [37]. Previous epidemiological research found that O1, O2, O18, and O78 accounted for more than 85% of *E. coli* in Eastern Chinese farms. The O-antigen is an essential element of the lipopolysaccharide (LPS) layer found in the outer membrane of *E. coli*. Similarly, a previous study reports serogroups from APEC strains, with O78 (16%) and O2 (10%) [54], which were slightly more prevalent compared to our results. Furthermore, another study reported for the first time that O145 may be emerging as a predominant serogroup of APEC in China, with a prevalence of (37.9%), which was higher than that of the other traditional APEC serogroups (O1 (4.7%), O2 (4.7%), O9 (7.1%), O21 (2.3%), O78 (16.7%)). The possible reason for its prevalence and oversight is the failure of vaccines that target the other major serogroups [26]. Similarly, a study reported O8 (37.6%) was the most prevalent serotype of APEC isolated from Wenchang chicken embryos, followed by O9 (16.9%), O102 (6.9%), O128 (3.8%), O21 (3%), and O78 (2.3%) [25]. Serotyping *E. coli* bacteria in isolated or diseased tissues is critical for disease diagnosis [55], epidemiology, and vaccine development [37].

The complex mechanism of biofilm formation encompasses several genes and regulatory networks. The initial stages of biofilm development, particularly within the first 12 h, are significantly influenced by adhesion genes such as *papC* and *tsh,* crucial for transcription and motility [56]. Previous research has examined the relationship between biofilm production and phylogenetic classifications. Notably, phylogroups B2 and D are frequently associated with enhanced biofilm formation, resistance to multiple drugs, a high capacity for iron uptake, and the presence of toxin-related genes [57].

In a previous epidemiological study, a substantial proportion of *E. coli* isolates derived from urine samples exhibited resistance to combinations of β-lactam antibiotics, β-lactamase inhibitors, quinolones, and cephalosporins, while showing low resistance levels to fosfomycin (2.7%), imipenem (3.2%), and meropenem (3.2%) [58]. The slight difference in our results is due to the frequent use of these antibiotics in poultry farming for therapeutic and prophylactic purposes. In these regions, antibiotics are often used without strict regulations, leading to overuse or misuse, which promotes the development of resistant strains. Furthermore, another study indicated that *E. coli* isolates demonstrated considerable antibiotic resistance, including quinolones, cephalosporins, aminoglycosides, carbapenems, and penicillin [59]. In Iran, research on uropathogenic *E. coli* strains revealed a predominant resistance to cefepime (100%) and cephalothin (74%), although these strains remained sensitive to imipenem (100%), vancomycin (100%), and doxycycline (100%) [60]. Additionally, a study conducted in Spain in 2022 collected 376 extraintestinal pathogenic *E. coli* strains, which exhibited high resistance rates to ciprofloxacin (48.7%), trimethoprim-sulfamethoxazole (47.9%), and ampicillin (38%) [18].

The capacity of isolates to form biofilms was also examined, as the biofilm-forming characteristics of Gram-negative bacteria play a crucial role in their virulence. Our findings reveal that 64 strains exhibited biofilm formation. A 2021 study assessed the biofilm formation capabilities of *E. coli* strains, reporting that 99% of the isolates demonstrated this ability [61]. Shah et al. identified that 50% of *E. coli* isolates derived from urine samples form biofilm [62]. Detho et al. also found that over half of the *E. coli* isolates demonstrate biofilms in vitro [63]. Another study with 126 *E. coli* isolates established that 80.2% could produce biofilms, with 42.1%, 16.7%, and 21.4% of biofilm producers categorized as weak, medium, and strong, respectively [64]. Additionally, a study explored the correlation between specific virulence factors and the biofilm-forming ability of extraintestinal pathogenic *E. coli*, concluding that 84.3% of the isolates were capable of biofilm formation. This elevated percentage was suggested to be associated with the virulence genes present in the strains [18]. Although *papC* and *tsh* are adhesion-related virulence genes believed to contribute to the early stages of biofilm formation, our findings indicate only a weak association between these genes and biofilm intensity. Specifically, the *tsh* gene exhibited a weak positive correlation with biofilm score (r = 0.155), while *papC* showed a weak negative correlation (r = −0.048). Previous studies have reported mixed findings regarding this association. Fattahi et al. demonstrated that *papC*-positive *E. coli* strains had a high capacity for biofilm production in urinary tract isolates [65]. Similarly, Laconi et al. found that ESBL/pAmpC-producing *E. coli* strains from broiler chickens frequently harbored virulence factors associated with enhanced biofilm formation [66]. Biofilms have the potential to develop on the surfaces of water systems, feeders, and drinking apparatus, serving as a continual source of bacterial contamination that proves challenging to eradicate through standard cleaning and disinfection techniques [67]. The ongoing presence of these pathogens can result in chronic infections among poultry and the transmission of zoonotic diseases [68]. The bacteria embedded within biofilms exhibit heightened resistance to antibiotics and disinfectants, complicating control efforts [64]. To address these challenges, farm management practices must incorporate regular cleaning, application of agents that disrupt biofilms, and ongoing monitoring of water and feed systems.

## 5. Conclusions

The present study reports a high prevalence of *E. coli* (68%) in colibacillosis-suspected tissue samples from commercial broiler farms in four Chinese provinces. The *E. coli* strains exhibited significant antibiotic resistance to most antibiotics. Furthermore, the molecular characterization of *E. coli* strains revealed that 52.9% carried at least one serotype and virulence gene. Similarly, 24.2% of the *E. coli* strains were classified as avian pathogenic *E. coli* due to the presence of five or more virulence genes, specifically *tsh*, *ompA*, *fimC*, *iss*, *fyuA*, and *astA*, in a single strain. Moreover, about half of the *E. coli* strains were identified as biofilm producers. Biofilm formation in *E. coli* may facilitate the development of more effective therapeutic strategies for managing infections. Biosecurity protocols play a crucial role in the management and prevention of colibacillosis. Implementing vaccination strategies aimed at particular *E. coli* serogroups, including O1, O2, O8, O9, O18, O21, O78, O102, O128, and O145, can markedly decrease the frequency of infections. Furthermore, alleviating stress through improved environmental conditions and upholding rigorous hygiene and sanitation standards in water supply systems and feeding apparatus are vital for reducing infection risks. When these biosecurity measures are followed, they can significantly enhance the control of colibacillosis in agricultural settings. However, this study has some limitations, particularly regarding the need for a more in-depth examination of the antibiotic resistance mechanisms exhibited by the strains. Further investigation into other genes that may influence biofilm formation is necessary. These findings can also contribute to essential insights into the virulence mechanisms of *E. coli* and support the formulation of more effective control strategies for this vital poultry pathogen.

## Figures and Tables

**Table 1:** Primer sequences and annealing temperatures were used for the PCR analysis of virulence-associated genes.

Gene	Description	Size (bp)	Primer Sequence (5′-3′)	Annealing Temperature (°C)	References
Serotypes					
*gnd*-F	Serotype O1	263	CGATGTTGAGCGCAAGGTTG	57	[27]
*rfb*O1-R			CATTAGGTGTCTCTGGCACG		
*rfb*O2-R	Serotype O2	355	GATAAGGAATGCACATCGCC		
*rfb*O18-R	Serotype O18	459	AGAAGCATTGAGCTGTGGAC		
*rfb*O78-R	Serotype O78	623	TAGGTATTCCTGTTGCGGAG		
O8-F O8-R	Serotype O8	448	CCAGAGGCATAATCAGAAATAACAG GCAGAGTTAGTCAACAAAAGGTCAG	53	[25,26]
O9-F O9-R	Serotype O9	1235	CGTCGGCAAGGCGTATAAATA CCCAGAAATCCATGCTC		
O21-F O21-R	Serotype O21	209	CTGCTGATGTCGCTATTATTGCTG TGAAAAAAAGGGAAACAGAAGAGCC		
O102-F O102-R	Serotype O102	1025	TCCGGTAAGTATCTTACGGCA GCACCAAATAGCGAAATACCA		
O128-F O128-R	Serotype O128	782	ATGATTTCTTACGGAGTGC CTCTAACCTAATCCCTCCC		
O145-F O145-R	Serotype O145	132	TTCGCGCACAGCATGGTTAT TACAATGCACCGCAAACAGT		
Virulence genes					
*iroN*	Iron acquisition	553	F: AATCCGGCAAAGAGACGAACCGCCT R: GTTCGGGCAACCCCTGCTTTGACTTT	63	[35]
*iutA*		302	F: GGCTGGACATCATGGGAACTGG R: CGTCGGGAACGGGTAGAATCG		
*ompT*	Protectins	496	F: TCATCCCGGAAGCCTCCCTCACTACTAT R: TAGCGTTTGCTGCACTGGCTTCTGATAC		
*ompA*		620	F: ATGATGGTCATCCGTCCCGT R: ATCAGTTCTGCAATAAATGC		
*iss*		323	F: CAGCAACCCGAACCACTTGATG R: AGCATTGCCAGAGCGGCAGAA		
*hlyE*	Toxins	450	F: GGCCACAGTCGTTTAGGGTGCTTACC R: GGCGGTTTAGGCATTCCGATACTCAG		
*astA*		116	F: TGCCATCAACACAGTATATCC R: TCAGGTCGCGAGTGACGGC	57	[36]
*papC*	Adhesins	501	F: TGATATCACGCAGTCAGTAGC R: CCGGCCATATTCACATAA	60	
*tsh*		824	F: ACTATTCTCTGCAGGAAGTC R: CTTCCGATGTTCTGAACGT		
*ibeA*	Invasins	171	F: AGGCAGGTGTGCGCCGCGTAC R: TGGTGCTCCGGCAAACCATGC	63	[35]
*iucD*	Aerobactin synthesis	613	F: GAAGCATATGACACAATCCTG R: CAGAGTGAAGTCATCACGCAC	54	[15,37]
*vat*	Vacuolating autotransporter toxin	939	F: TCCATGCTTCAACGTCTCAGAG R: CTGTTGTCAGTGTCGTGAACG		
*cvi/cva*	Structural genes of colicin V operon	598	F: TCCAAGCGGACCCCTTATAG R: CGCAGCATAGTTCCATGCT	57	
*fimC*	Type 1 fimbriae (D-mannose-specific adhesin)	513	F: TATGTTGGCTTTGAAATGGG R: ATCCAGAGCAGCCTGACCTT	63	
*fyuA*	Ferric yersinia uptake	209	F: GGCGGCGTGCGCTTCTCGCA R: CGCAGTAGGCACGATGTTGTA		

**Table 2:** Generalized linear model (GLM) estimates for the effect of province on *E. coli* prevalence in broiler tissue samples.

Province	Coefficient	Std. Error	z	*p*-Value
Liaoning	−0.178	0.423	−0.422	0.673
Shandong	−0.348	0.419	−0.832	0.405
Xinjiang	+0.633	0.466	+1.358	0.174

**Table 3:** Prevalence of *E. coli* serotypes in different regions.

Serotype	Shandong (*n* = 34)	Fujian (*n* = 30)	Liaoning (*n* = 32)	Xinjiang (*n* = 40)	Positive
*rfb*O1	2 (5.9%) ^ns^	4 (13.3%) ^ns^	2 (6.3%) ^ns^	2 (5.0%) ^ns^	10 (7.4%)
*rfb*O2	5 (14.7%) ^ns^	2 (6.7%) ^ns^	2 (6.3%) ^ns^	3 (7.5%) ^ns^	12 (8.8%)
O8	5 (14.7%) ^ns^	3 (10.0%) ^ns^	4 (12.5%) ^ns^	5 (12.5%) ^ns^	17 (12.5%)
O9	4 (11.8%) ^ns^	4 (13.3%) ^ns^	3 (9.4%) ^ns^	4 (10.0%) ^ns^	15 (11.0%)
*rfb*O18	3 (8.8%) ^ns^	4 (13.3%) ^ns^	4 (12.5%) ^ns^	3 (7.5%) ^ns^	14 (10.3%)
O21	2 (5.9%) ^ns^	1 (3.3%) ^ns^	1 (3.1%) ^ns^	2 (5.0%) ^ns^	6 (4.4%)
*rfb*O78	2 (5.9%) ^ns^	4 (13.3%) ^ns^	3 (9.4%) ^ns^	2 (5.0%) ^ns^	11 (8.1%)
O102	3 (8.8%) ^ns^	3 (10.0%) ^ns^	3 (9.4%) ^ns^	7 (17.5%) ^ns^	16 (11.8%)
O128	2 (5.9%) ^ns^	-	5 (15.6%) ^ns^	6 (15.0%) ^ns^	13 (9.6%)
O145	6 (17.6%) ^ns^	5 (16.7%) ^ns^	5 (15.6%) ^ns^	6 (15.0%) ^ns^	22 (16.2%)

^ns^ indicates a non-significant difference between the rows.

**Table 4:** Prevalence of virulence genes in *E. coli* strains isolated from four regions of China.

Virulence Genes	Xinjiang (*n* = 40)	Shandong (*n* = 34)	Liaoning (*n* = 32)	Fujian (*n* = 30)	Total Positive (*n* = 136)
*ompA*	3 (7.5%) ^ns^	3 (8.8%) ^ns^	3 (9.3%) ^ns^	2 (6.6%) ^ns^	11 (8%)
*astA*	4 (10%) ^ns^	2 (5.8%) ^ns^	2 (6.2%) ^ns^	2 (6.6%) ^ns^	10 (7.3%)
*iss*	4 (10%) ^ns^	3 (8.8%) ^ns^	3 (9.3%) ^ns^	2 (6.6%) ^ns^	12 (8.8%)
*ompT*	1(2.5%) ^ns^	1 (2.9%) ^ns^	1 (3.1%) ^ns^	1 (3.3%) ^ns^	4 (2.9%)
*iroN*	3 (7.5%) ^ns^	1 (2.9%) ^ns^	1 (3.1%) ^ns^	1 (3.3%) ^ns^	6 (4.4%)
*hlyE*	2 (5%) ^ns^	3 (8.8%) ^ns^	2 (6.2%) ^ns^	1 (3.3%) ^ns^	8 (5.8%)
*iutA*	2 (5%) ^ns^	2 (5.8%) ^ns^	3 (9.3%) ^ns^	1 (3.3%) ^ns^	8 (5.8%)
*papC*	2 (5%) ^ns^	3 (8.8%) ^ns^	3 (9.3%) ^ns^	3 (10%) ^ns^	11 (8%)
*tsh*	3 (7.5%) ^ns^	4 (11.7%) ^ns^	4(12.5%) ^ns^	3 (10%) ^ns^	14 (10.2%)
*ibeA*	4 (10%) ^ns^	2 (5.8%) ^ns^	1 (3.1%) ^ns^	3 (10%) ^ns^	10 (7.3%)
*iucD*	2 (5%) ^ns^	1 (2.9%) ^ns^	1 (3.1%) ^ns^	3 (10%) ^ns^	7 (5.1%)
*vat*	3 (7.5%) ^ns^	2 (5.8%) ^ns^	2 (6.2%) ^ns^	1 (3.3%) ^ns^	8 (5.8%)
*cvi/cva*	1(2.5%) ^ns^	1 (2.9%) ^ns^	1 (3.1%) ^ns^	3 (10%) ^ns^	6 (4.4%)
*fimC*	3 (7.5%) ^ns^	2 (5.8%) ^ns^	3 (9.3%) ^ns^	2 (6.6%) ^ns^	10 (7.3%)
*fyuA*	3 (7.5%) ^ns^	4 (11.7%) ^ns^	2 (6.2%) ^ns^	2 (6.6%) ^ns^	11 (8%)

^ns^ indicates a non-significant difference between the columns.

## Data Availability

The original contributions presented in this study are included in the article/Supplementary Materials. Further inquiries can be directed to the corresponding authors.

## Supplementary Materials

The following supporting information can be downloaded at: https://www.mdpi.com/article/10.3390/microorganisms13051017/s1, Figure S1: Molecular identification of *E. coli*; Figure S2: Molecular detection rate of *E. coli* virulence genes and serotypes; Figure S3: Antibiotic susceptibility profile of *E. coli* strains.

## Abbreviations

The following abbreviations are used in this manuscript:

PB	Polymyxin B
A/C	Amoxicillin/Clavulanate
CAZ	Ceftazidime
SXT	Sulfamethoxazole/Trimethoprim
ENF	Enrofloxacin
CP	Cefepime
AMP	Ampicillin
KAN	Kanamycin
STR	Streptomycin
MEM	Meropenem
FFC	Florfenicol
CLM	Clindamycin
TET	Tetracycline
