# Supplementary material for: Molecular Characterization, Antibiotic Resistance, and Biofilm Formation of Escherichia coli Isolated from Commercial Broilers from Four Chinese Provinces
Source: Microorganisms. 2025 Apr 28;13(5):1017. doi: 10.3390/microorganisms13051017 (PMC12113953; doi:10.3390/microorganisms13051017)
Supplement: Supplementary file 1 [file microorganisms-13-01017-s001.zip › microorganisms-3622147-supplementary.pdf]

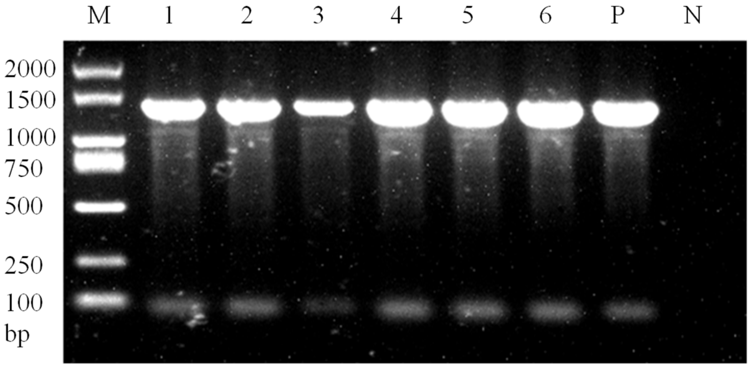

Figure S1: Representative gel electrophoresis picture of *E. coli*-positive samples. Lane 1-6: *phoA* gene positive (1416 bp), M: DL2000 Plus DNA Marker (Vazyme), P: Positive control, N: Negative control.

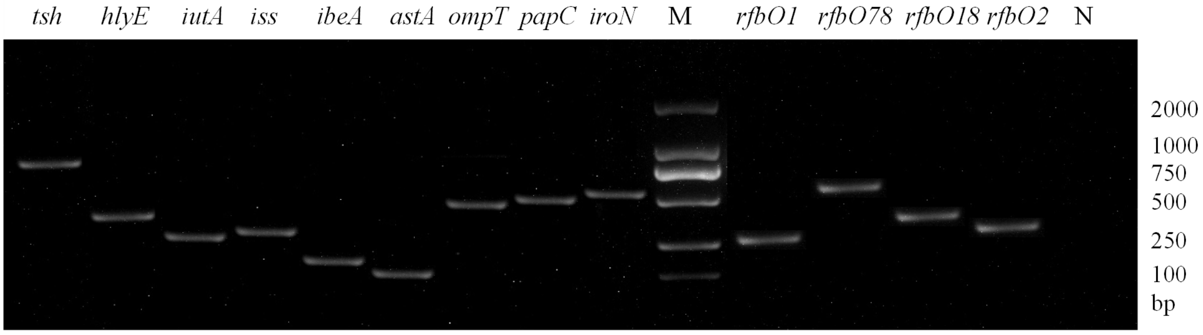

Figure S2: The figure represents the virulence genes and serotypes detected by PCR: *tsh* gene (824 bp), *hlyE* (450 bp), *iutA* (302 bp), *iss* (323 bp), *ibeA* (171 bp), *astA* (116 bp), *ompT* (496 bp), *papC* (501 bp), *iroN* (553), *rfbO1* gene (263 bp), *rfbO78* gene (623 bp), *rfbO18* gene (459 bp), and *rfbO2* (355 bp). M: DL2000 Plus DNA Marker (Vazyme). N: Negative control.

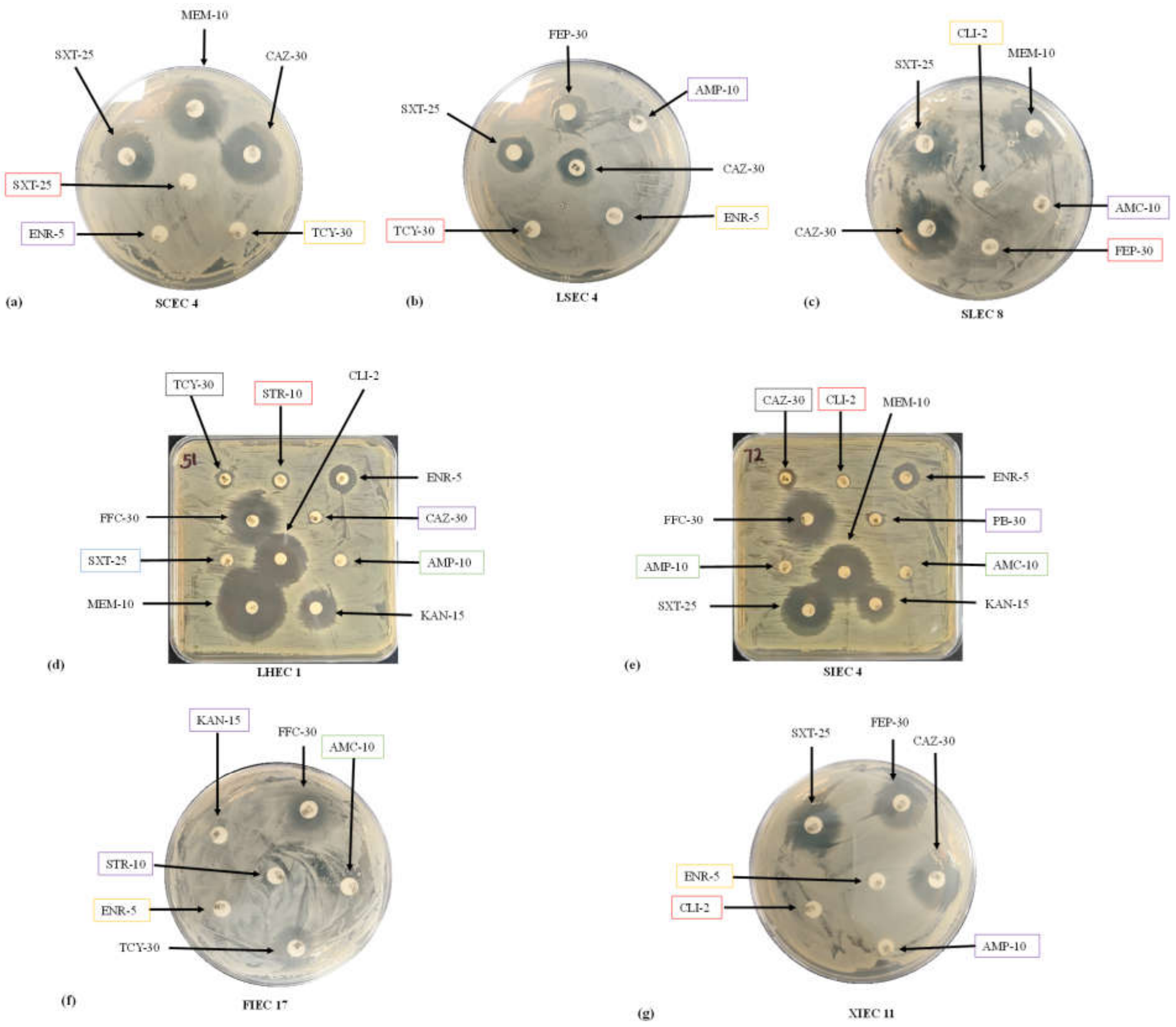

Figure S3: Antibiotic susceptibility testing of *E. coli* isolates: (a) Shandong Crop *E. coli* (SCEC 4) (b) Liaoning Spleen *E. coli* (LSEC 4), (c) Shandong Liver *E. coli* (SLEC 8), (d) Liaoning Heart *E. coli* (LHEC 1), (e) Shandong Intestine *E. coli* (SIEC 4), (f) Fujian Intestine *E. coli* (FLIEC 17), and (g) Xinjiang Intestine *E. coli* (XIEC 11).
